# SPOP promotes cervical cancer progression by inducing the movement of PD-1 away from PD-L1 in spatial localization

**DOI:** 10.1186/s12967-022-03574-6

**Published:** 2022-08-30

**Authors:** Jiangchun Wu, Yong Wu, Qinhao Guo, Siyu chen, Simin Wang, Xiaohua Wu, Jun Zhu, Xingzhu Ju

**Affiliations:** 1 Department of Oncology, Shanghai Medical College, Fudan University, Shanghai, 200032 China; 2 Department of Gynecologic Oncology, Fudan University Shanghai Cancer Center, Fudan University, Shanghai, 200032 People’s Republic of China

**Keywords:** Cervical cancer, SPOP, Multiplex immunofluorescence, PD-1/PD-L1 axis, CXCL16/CXCR6 axis

## Abstract

**Background:**

Metastasis is a major obstacle in the treatment of cervical cancer (CC), and SPOP-mediated regulatory effects are involved in metastasis. However, the mechanisms have not been fully elucidated.

**Methods:**

Proteomic sequencing and SPOP immunohistochemistry (IHC) were performed for the pelvic lymph node (pLN)-positive and non-pLN groups of CC patients. The corresponding patients were stratified by SPOP expression level for overall survival (OS) and relapse-free survival (RFS) analysis. In vitro and in vivo tests were conducted to verify the causal relationship between SPOP expression and CC metastasis. Multiplex immunofluorescence (m-IF) and the HALO system were used to analyse the mechanism, which was further verified by in vitro experiments.

**Results:**

SPOP is upregulated in CC with pLN metastasis and negatively associated with patient outcome. In vitro and in vivo, SPOP promotes CC proliferation and metastasis. According to m-IF and HALO analysis, SPOP may promote CC metastasis by promoting the separation of PD-1 from PD-L1. Finally, it was further verified that SPOP can achieve immune tolerance by promoting the movement of PD-1 away from PD-L1 in spatial location and function.

**Conclusion:**

This study shows that SPOP can inhibit the immune microenvironment by promoting the movement of PD-1 away from PD-L1, thereby promoting pLN metastasis of CC and resulting in worse OS and RFS.

## Highlights

1. The SPOP is associated with pelvic lymph node (pLN) metastasis and prognosis in cervical cancer (CC) patients.
2. This paper discusses the potential mechanism of pLN metastasis of CC from the perspective of spatial location.
3. This is a multi-cross study, including clinical data, tissue microarray (TMA), multicolor immunofluorescence (m-IF), spatial immunolocalization, in vitro and in vivo functional and mechanism research fusion, from clinical to basic and clinical research.

## Background

Cervical cancer (CC) is the fourth most common cancer and the third leading cause of cancer-related death in women globally [1]. Metastasis, especially pelvic lymph node (pLN) metastasis, is a major challenge for CC treatment, as most CC-related mortality is caused by metastasis [2–5]. With the progression of diagnostic technology and therapeutic methods, the clinical outcomes of CC have significantly improved [5–8]; however, there are currently no effective treatment options for preventing or inhibiting CC metastasis, in part because the molecular mechanisms that underlie CC invasion and pLN metastasis have not been fully elucidated [9].

Nuclear speckle-type pox virus and zinc finger protein (SPOP), a representative substrate-recognition subunit (SRS) of cullin-RING E3 ligase 3 (CRL3, a member of the CRL complex family), has been recognized to play a dual role in the development and progression of human cancers, including lung, colon, gastric, prostate and liver cancers [10–16]. However, studies focusing on the role of SPOP in the development of CC are still lacking. In 2007, Byun et al. demonstrated that SPOP confers a proapoptotic function in HeLa cells [17]. Recently, Pang et al. demonstrated that the CUL3/SPOP E3 ubiquitin ligase degraded DRAK1, thus promoting paclitaxel-resistant CC cell growth [18].

Traditional procedures have been used to elucidate the mechanism by regularly exploring molecular pathways [19–21]. However, due to the tissue-destructive nature of most of these methods, the spatial distribution and temporal distribution of the immune milieu in situ are not preserved [22]. Multiplex immunohistochemistry/immunofluorescence (m-IHC/IF) has emerged and allows for high-throughput multiplex staining and further standardized quantitative analysis, resulting in highly efficient, reproducible, and cost-effective tissue studies [22–26]. This is a multilabel immunofluorescence staining method derived from tyramine signal amplification (TSA) technology, which can recolour more than 7 antigens on the same tissue section samples and perform differentiated labelling [27]. Afterwards, HALO (Indica Labs, Albuquerque, USA), an image analysis system, not only can be used for quantitative tissue analysis but can also reveal the spatial location of each target [28–32]. The HALO system was used to segment each point on each tissue chip according to different cases, and then quantitative pathological analysis was conducted by whole- slide imaging (WSI) of each staining target on the whole TMA. From this data, we can obtain the area of each point site, the number of targets, and the spatial distance of each target [22]. This will enable us to study the mechanism of tumorigenesis and development from the perspective of spatial location and expand the depth of our research.

The programmed cell death protein 1 (PD-1) is one of the co-inhibitory immune checkpoint receptors induced upon T cell activation [33–35]. Through transducing negative signaling of effector T cell activity by the interaction with programmed death-ligand 1 (PD-L1), PD-1 serves as a mediator for tumour cells to survive by escaping T cell killing [36, 37]. In addition, high numbers of PD-1 positive immune infiltrates are associated with significantly increased disease-free survival [35]. Therefore, the PD-1/PD-L1 axis plays a crucial role in the immune microenvironment of tumours.

In this study, we show that overexpression of SPOP is associated with pLN metastasis and clinical outcomes by inhibiting the immune microenvironment by promoting the movement of PD-1 away from PD-L1. This is the first study to explore the potential mechanism of CC pLN metastasis from the spatial relationship between molecules. Exploring the mechanism of pLN metastasis in CC will provide a new direction for the future treatment of CC.

## Methods

### Patient cohort

Written informed consent was obtained from all patients before sample collection. All procedures were approved by the institutional Ethics Review Committee of the World Health Organization of the Collaborating Center for Research in Human Production and authorized by the Human Ethics Committee of Fudan University Shanghai Cancer Center (FUSCC).

A retrospective cohort study was conducted in the Department of Gynecology Oncology, FUSCC, and included 180 patients with 2009 FIGO stage IB1-IIA2 disease who underwent radical abdominal hysterectomy with or without bilateral salpingo-oophorectomy and pelvic ± para-aortic lymphadenectomy from 2009 to 2012. For all the enrolled patients, an experienced gynaecological oncologist performed standard pelvic lymphadenectomy. All the microscopic slides, including those from gene SPOP staining, were reviewed and graded by the same professional gynaecologic pathologist and were reconfirmed by another experienced gynaecologic pathologist. All clinical records were retrospectively studied.

### Proteomic sequencing and data processing

Proteomic sequencing was performed in 5 pLN-positive CC tissues and 5 pLN-negative CC tissues.

The project was divided into a pre-experiment and a formal experiment. The preexperiment included protein extraction, protein quantification, SDS-PAGE, protein enzymatic hydrolysis, liquid chromatography (LC)-mass spectrometry (MS)/MS analysis, database query, and quality control [38–40]. The formal experiment was carried out based on the preexperiment. The samples qualified by quality control in the preexperiment were formally tested by high-resolution MS to obtain the original MS data.

In the process of label-free project data analysis, database query and result evaluation are usually carried out on the original MS data, and subsequent information analysis is carried out on the qualified data of quality control, including identification and screening of trusted peptides and proteins, quantitative analysis of proteins and screening of differentially expressed proteins [41, 42].

### Immunohistochemical staining (IHC)

IHC of SPOP was performed on paraffin-embedded sections of 180 CC tissues. The slice thickness was set at 5 µm, and 3 sections were selected from each specimen. Slides were rinsed and incubated with primary antibodies against SPOP (1:100; Cell Signaling Technology; CST). Subsequent antibody detection was carried out with a Cy3-conjugated goat anti-rabbit secondary antibody (1:300; Invitrogen). The expression level of SPOP was determined by the immunoreactive score (IRS) [43–45].

### Cell culture and reagents

HeLa, SiHa, ME-180, and MS751 human CC cell lines were acquired from the American Type Culture Collection (ATCC) and authenticated by short tandem repeat profiling. These cell lines were cultured at 5% CO_2_ and 37 °C in high-glucose Dulbecco’s modified Eagle’s medium (DMEM; Gibco, USA) containing 10% foetal bovine serum (FBS; Gibco, USA) and 1% penicillin–streptomycin (Gibco, USA).

### Plasmids

The SPOP overexpression plasmid was constructed by cloning the cDNA into the PGMLV-CMV-EF1-ZsGreen1-T2A-Puro vector (System Biosciences, CA, USA). Plasmids carrying shRNAs targeting SPOP were generated using the U6-MCS-CMV-ZsGreen1-PGK-Puro vector (System Biosciences, CA, USA). The siRNAs and matched empty vector controls were obtained from Lncbio (Shanghai, China). The SPOP shRNA target sequence was as follows: shRNA1: GTAGCACCAACTCTCAGCTA, shRNA2: CCTCCGGCAGAAATGTCGAG, shRNA3: TGACTTCACCCATTTCCTCC.

### RNA extraction and qRT–PCR

Total RNA was extracted from samples and cells using TRIzol reagent (Life Technologies, CA, USA) according to the manufacturer’s protocol. qRT–PCR was conducted using TB Green PCR Master Mix (TaKaRa, Dalian, China) in an ABI7900HT Real-Time PCR system (Applied Biosystems, USA). The relative quantification was normalized to β-actin with the 2-cycle threshold (CT) formula. The primers used for qRT–PCR are listed as follows: β-actin-F: AATGGACTATCATATGCTTACCGTAACTTGAAAGTATTTCG; β-actin-R: CTTTAGTTTGTATGTCTGTTGCTATTATGTCTACTATTCTTTCC; and SPOP-F: GCCCCGTAGCTGAGAGTTG; SPOP-R: ACTCGCAAACACCATTTCAGT.

### Western blot (WB) analysis

WB was performed as previously described [46]. The cells were sonicated in lysis buffer containing protease inhibitor cocktail for 15 min (min). Then, the lysates were centrifuged (12,000 *g*, 15 min, 4 ℃) to collect the supernatants. The Bio-Rad Protein Assay Kit (Hercules, CA, USA) was used to quantify the protein concentration. Approximately 25–30 µg of protein from each sample was separated by 10% SDS-PAGE and electroblotted onto a polyvinylidene fluoride membrane. After blocking with 5% bovine serum albumin (BSA), the membranes were incubated individually overnight at 4 ℃ with the corresponding primary antibodies (rabbit monoclonal anti-SPOP antibody, 1:1000, CST; mouse monoclonal anti-COL6A3, 1:5000, Abcam; mouse monoclonal anti-CXCL16, 1:2000, Santa Cruz; rabbit monoclonal anti-⍺SMA, 1:2000, Abcam; mouse monoclonal anti-β-actin, 1:5000, Abcam, Inc.). After washing three times with phosphate-buffered saline (PBS) for 10 min, the membranes were incubated with the secondary antibody, either horseradish peroxidase-conjugated anti-rabbit or anti-mouse IgG, for 2 h at room temperature (RT). Finally, the membranes were developed using an enhanced chemiluminescent analysis system (Super Signal West Pico Chemiluminescent Substrate, Pierce) and exposed to X-ray film. The grey value was analysed by ImageJ software.

### CCK-8 assay

Cells were seeded and cultured in 96-well plates overnight. At 1, 2, 3, 4, and 5 days, 10 µl cell counting kit-8 solution (MedChem Express, Monmouth Junction, NJ, USA) was added to each well, followed by further incubation at 37 °C. After 1 h, the absorbance was measured at 450 nm wavelength to assess cell proliferation ability [47].

### Colony-formation assay

Cells were seeded and cultured in 6-well plates at a density of 1 × 10^3^ cells/well. During this period, the medium was changed as needed. After two weeks, the cells were fixed with 4% paraformaldehyde (PFA, Sangon Biotech) and stained with 0.1% crystal violet (Beyotime) for 15 min [48].

### Cell cycle assay

Cells were seeded and cultured in 6-well plates at a density of 3 × 10^5^ cells/well. After 2 days, cells for cell cycle analysis were digested with trypsin (HyClone), washed with PBS three times, and fixed in 70% ethanol overnight at − 20 ℃. Then, the cells were centrifuged at 500 × *g* for 20 min and washed three times with cold PBS. They were then treated with RNase A (0.1 mg/ml) and propidium iodide (PI, 0.05 mg/ml) for 15 min in the dark. Finally, flow cytometry was applied [49].

### Cell migration and invasion assays

For wound-healing assays, cells were cultured in 6-well plates and scratched with a 1 ml pipette tip, and the wounds were photographed at 0 h and 36 h. The relative migration ratio was calculated [50, 51].

For migration assays, a 24-well plate with Transwell chambers (8 µm pore size, Coring) was applied. A serum concentration difference was formed between the upper and lower chambers (upper: 10% FBS; lower: FBS-free). A total of 4 × 10^4^ cells were cultured in the upper chamber containing solubilized extracellular matrix (ECM)-coated members, as previously described in the manufacturer’s instructions (Coring Matrigel invasion assay; USA), for 24 h. The cells on the lower surface of the chambers were fixed with 4% paraformaldehyde (PFA) for 15 min and stained with 0.1% crystal violet for 15 min [52, 53].

### Animal imaging

BALB/c nude mice (females aged 4–5 weeks, 18–20 g) were purchased from the Shanghai Family Planning Institute (Shanghai, China) and housed under standard specific pathogen-free (SPF) conditions in the animal room of Shanghai Medical College of Fudan University. To develop subcutaneous tumour and metastasis models, a total of 5 × 10^6^ SPOP-knockdown and vector-transfected HeLa cells were suspended in 0.1 ml of sterile PBS and subsequently randomly injected into the flanks or the vaginal wall (n = 4 per group). Four weeks later, subcutaneous tumour volume was measured every 7 days after the appearance of tumours, and tumour volume was calculated as (length*width^2^) * 0.5. For the metastatic tumour model, F18-FDG of the same volume was injected through the tail vein. Half an hour later, micropositron emission tomography-computed tomography (microPET-CT) imaging was performed on the animals. Finally, the activity of F18-FDG injected into animals was calculated by measuring the activity of nuclides before injection and after injection, and the standard uptake value (SUV) was obtained.

### Making tissue microarray (TMA)

The 180 CC patients’ tissues (in situ) were prepared into TMA as previously described [54]. We used paraffin-embedded tissue chips. Dozens to hundreds of small cylindrical tissues were collected from numerous tissue wax blocks (the donor wax block) by the method of fine needle drilling of a tissue chip making machine, and they were arranged neatly into another empty white wax block (the recipient wax block) to make a tissue chip wax block. Then, the tissue chip wax block was sliced, and the slice was transferred to slides to make TMAs.

The detailed steps are as follows: First, the recipient wax block was made with a size of 45 mm × 20 mm. Holes with a spacing of 0.1 mm and a diameter of 0.6 mm were punched on the wax block, and the coordinates of each hole were accurately positioned. Second, representative points on the sample wax block were marked according to HE staining, including pLN-positive CC tissue and pLN-negative CC tissue. The corresponding points on the wax block tissue were selected and punctured with a stainless-steel needle (inner diameter: 0.6 mm; depth: 2–3 mm), which was fixed in the holes of the recipient wax block. Finally, the recipient wax block was precooled at 4 ℃ for approximately 4 h, and a microtome was used to correct the whole tissue. Then, 30–50 slices with a thickness of 5 µm were quickly generated and applied to the slides.

### M-IF staining protocol

An Opal 7-colour kit (NEL811001KT, PerkinElmer) was used for mIF. TMAs were dewaxed and rehydrated. In the first step, the antigen was retrieved at 125 ℃ for 3 min and then cooled to room temperature (RT). The membranes were washed with TBST three times for 5 min and incubated in H_2_O_2_ for 10 min. The membranes were repeatedly washed and blocked with blocking buffer. The primary antibody, PD-L1 (ab237726, Abcam, 1:500, dye 480), was incubated at RT for 30 min. Slides were washed, and an HRP-conjugated secondary antibody was incubated at RT for 10 min. TSA dye (1:100) was applied for 10 min after washing. The procedures were repeated six times using the following antibodies: CD3 (ab16669, Abcam, 1:200, dye 690; used as T lymphocyte cell marker), CD8 (ab93278, Abcam, 1:100, dye 570; used as cytotoxic T-cell marker), CD56 (ab75813, Abcam, 1:500, dye 620; used as NK-cell marker), CD68 (ab213363, 1:1000, Abcam, dye 780; used as panmacrophage marker), programmed death-1 (PD-1) (ab237728, Abcam, 1:300, dye 520), and programmed death ligand-1 (PD-L1) (ab237726, 1:500, dye 480). Anti-mouse (NEF822001EA, PerkinElmer) or anti-rabbit (NEF812001EA, PerkinElmer) secondary antibodies were used at a 1:1000 dilution [22, 55, 56].

With further analysis by the HALO system, we quantified the number of six immune targets and the spatial position relationship between them [56].

### Coimmunoprecipitation (Co-IP)

Cells were washed three times with PBS and lysed in IP buffer (NP40 buffer 890 µl, 10% NP40 100 µl, 10 µl protease inhibitors) (Shanghai Shenger Biotechnology). Protein concentrations were quantified by BCA assay and boiled in each sample with 5× loading buffer at 100 °C. For the IP assay, plasmids were transfected using Myc-SPOP in HeLa cells, and after 42 h, cells were harvested and lysed in IP buffer. Whole-cell extracts were incubated thoroughly on a Ferris wheel with the corresponding primary antibodies overnight at 4 °C. Antibody-bound proteins were precipitated with magnetic beads (Shanghai Shenger Biotechnology) according to the manufacturer’s protocol. The beads were washed three times with lysis buffer and then eluted in 2X SDS sample loading buffer in a 100 °C metal bath. Eluted proteins were then separated by SDS–polyacrylamide gel electrophoresis, transferred to NC membranes (Millipore), and detected using corresponding primary antibodies coupled with a horseradish peroxidase-conjugated secondary antibody by chemiluminescence (GE Healthcare). The Co-IP proteins were used for MS (Jikai Biotechnology, China) or WB analysis.

### Half maximal inhibitory concentration (IC50)

Cells (4 × 10^3^ cells/well) were seeded and cultured in 96-well plates overnight. At 1 day, PD-1 was treated with different concentrations (0, 0.0625 mg/ml, 0.125 mg/ml, 0.25 mg/ml, 0.5 mg/ml, 1 mg/ml, 2 mg/ml, 4 mg/ml, 8 mg/ml). At 2 days, 10 µl of cell counting kit-8 solution (MedChem Express, Monmouth Junction, NJ, USA) was added to each well, followed by further incubation at 37 °C. After 1 h, the absorbance was measured at 450 nm wavelength to assess cell proliferation ability [47].

### Statistical analysis

Statistical analysis was performed with IBM SPSS 23.0 (Chicago, IL, USA), GraphPad Prism 8, and R language. Comparisons between two conditions were based on a two-sided Student’s test. The results of all statistical analyses were reported as p values from two-tailed tests, and *P* < 0.05 was judged to be statistically significant (*P < 0.05, **P < 0.01, and ***P < 0.001).

## Results

### Patient characteristics

The study cohort included 180 cases of CC with high-quality TMA. The median age of the patients was 46 years (95% confidence interval (CI) 23–71 years). The histological diagnosis was squamous cell carcinoma in all cases, and based on the FIGO 2009 guidelines, thirty-nine (43.3%) subjects were stage IB, and fifty-one (56.7%) subjects were stage IIA. The median follow-up time was 123 months (95% CI 107.28–138.72 months); seven (7.8%) subjects died and thirteen (14.4%) subjects relapsed.

### SPOP is frequently upregulated in CC patients with pLN metastasis and is negatively associated with patient outcome

**Fig. 1:**
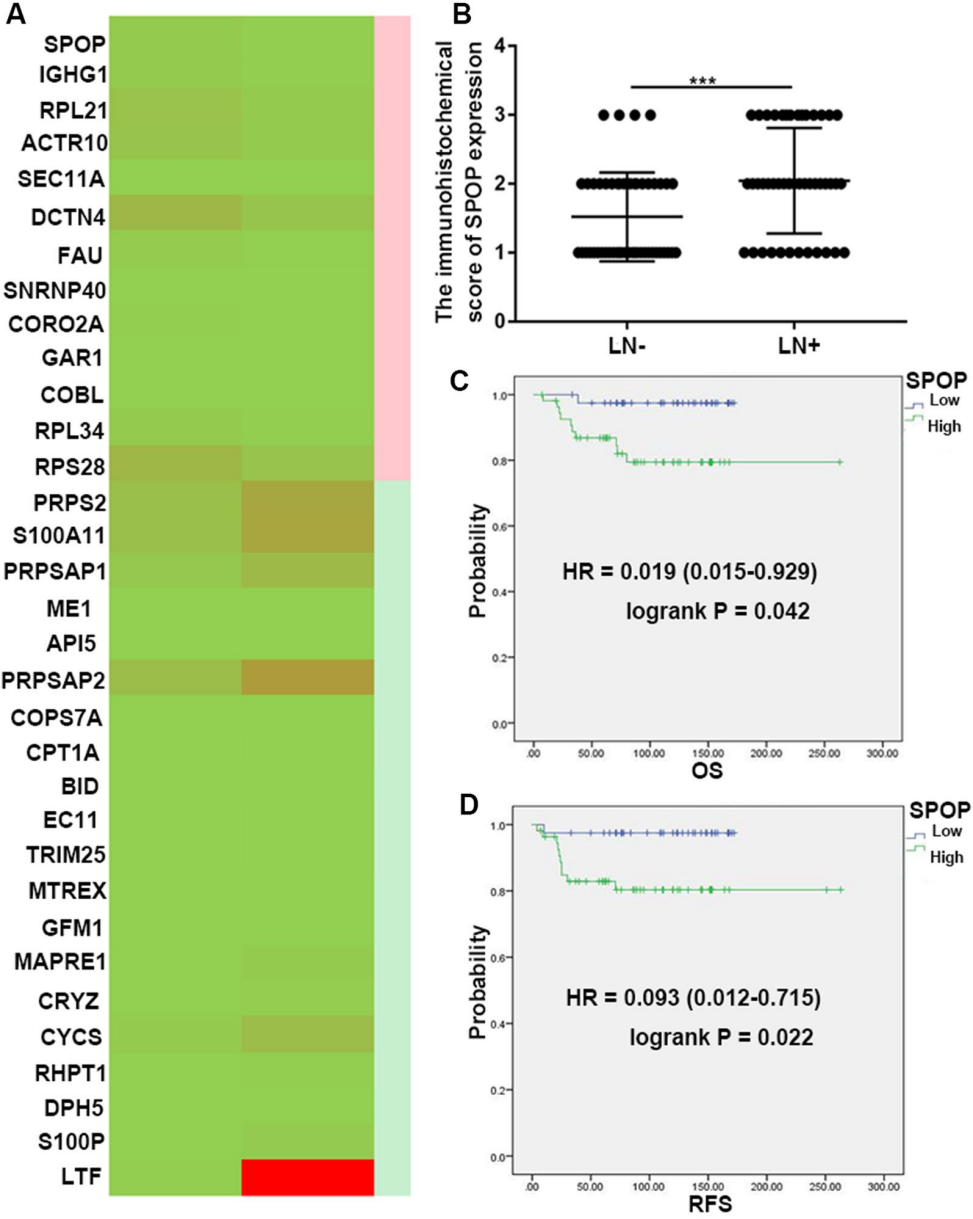
SPOP is upregulated in CC patients with pLN metastasis and negatively associated with patient outcome. **A** The expression of 33 key proteins in our proteomic sequencing data from 5 pLN-positive CC tissues and 5 pLN-negative CC tissues are presented in the heatmap. **B** The IHC scores of SPOP in the pLN-positive CC group were significantly higher than those in the pLN-negative CC group (*P* < 0.01). **C**, **D** Kaplan–Meier analysis was performed to assess the associations between SPOP expression and the OS (*P* = 0.042) and RFS (*P* = 0.022) of patients with CC. Data are presented as the mean ± SEM

We analysed the expression of SPOP in CC with pLN metastasis or without pLN metastasis in our centre. Through proteome sequencing of the two groups, we found that the SPOP protein was significantly increased in the positive group (|FC|> 2.5) (Fig. 1A). Additionally, the SPOP IHC scores (IHC score 1: Low group; IHC score 2 and 3: High group) reached 1.52 ± 0.091 in the pLN-negative group and 2.04 ± 0.12 in the pLN-positive group (pLN-negative versus pLN-positive, *P* < 0.01) (*P* = 0.0005) (Fig. 1B). The above data suggest that SPOP may promote pLN metastasis in CC.

Furthermore, we applied the information of our centre to group the patients by SPOP expression level. High SPOP levels (score 3) were markedly associated with worse overall survival (OS; HR = 0.019 (0.015–0.029), *P* = 0.042) and relapse-free survival (RFS; HR = 0.093 (0.012–0.715), *P* = 0.022) (Fig. 1C, D). These data suggest that higher SPOP expression will result in considerably worse OS and RFS in CC patients.

### SPOP promotes CC cell proliferation in vitro

**Fig. 2:**
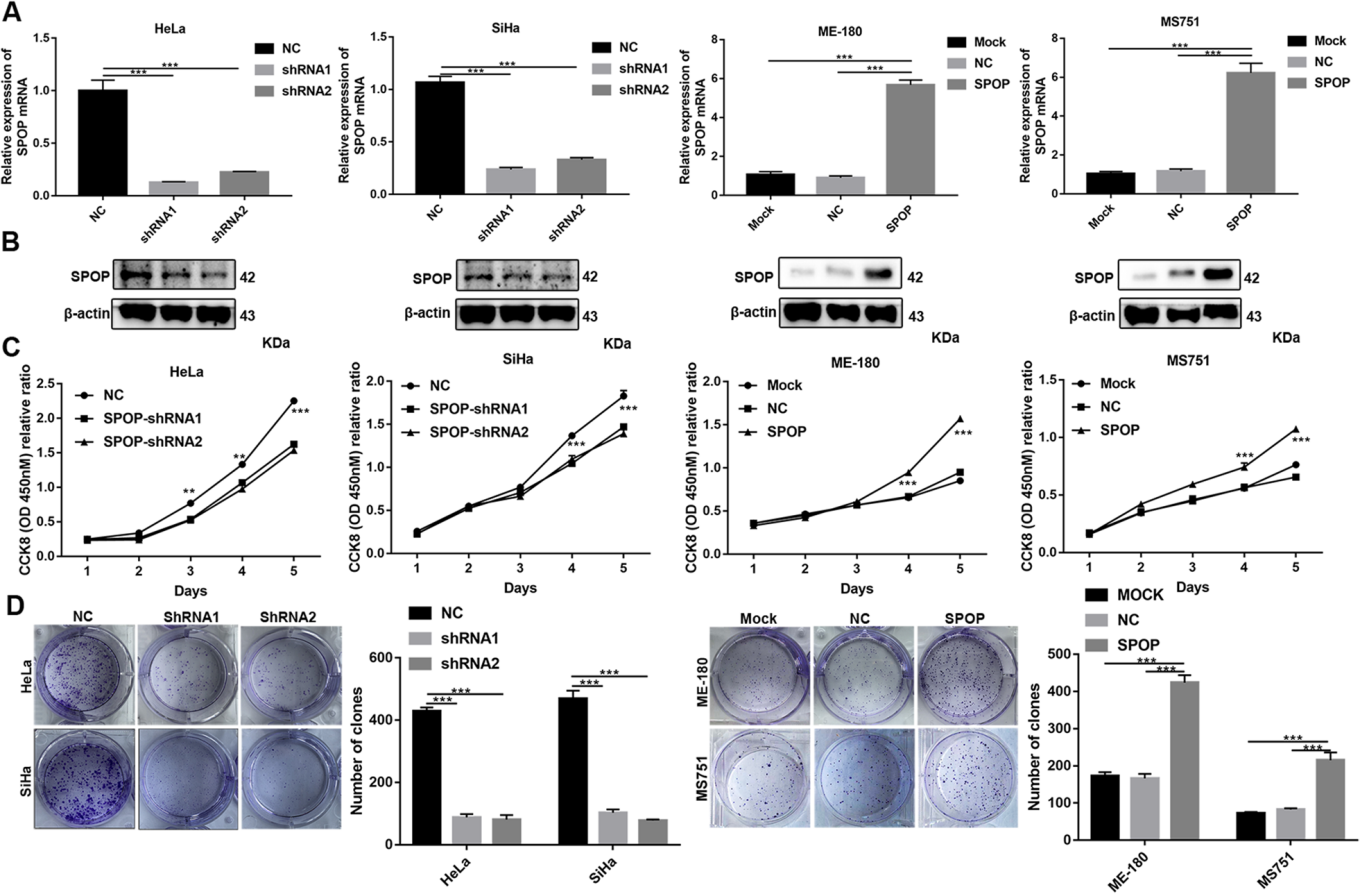
Effects of SPOP on the proliferation of CC cells in vitro. **A** qRT–PCR and **B** Western blot analyses of SPOP levels following SPOP knockdown and overexpression. **C** CCK-8 and **D** colony formation assays were performed to assess the changes in proliferation

First, we established stable cell lines by knocking down or overexpressing SPOP. Verification was then performed at the protein and RNA levels (Fig. 2A, B).

To further determine the tumour-promoting effect of SPOP on cell proliferation, we measured the expression levels of SPOP in various CC cell lines. Based on the high endogenous expression of SPOP in HeLa and SiHa cells, we designed and synthesized two independent small hairpin RNAs (shRNAs) to effectively reduce the SPOP RNA level, whereas ME-180 and MS751, with low endogenous SPOP expression levels, were transfected with lentivirus containing the SPOP sequence within the PGMLV vector to generate stable overexpressed cell lines. As shown in the results, SPOP knockdown significantly suppressed HeLa and SiHa cell proliferation, whereas SPOP overexpression significantly promoted ME-180 and MS751 cell proliferation (Fig. 2C). In addition, knockdown or overexpression of SPOP significantly decreased or increased, respectively, the colony formation of CC cells (Fig. 2D).

**Fig. 3:**
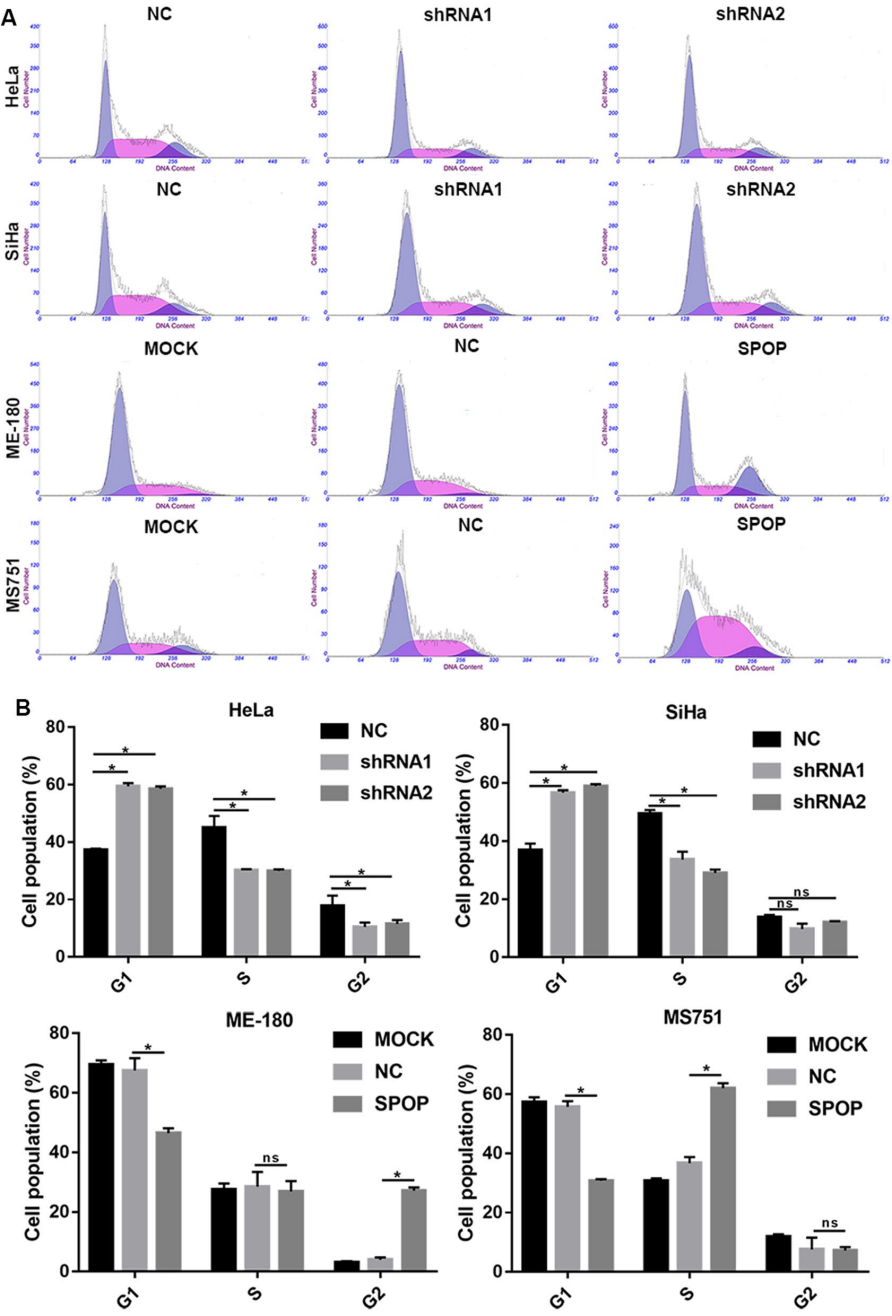
In vitro cell cycle assay. **A** The diagram of cell cycle. **B** In HeLa cells, cell cycle experiments showed that SPOP knockdown could significantly promote the proportion of G1 phase cells and reduce the proportion of S and G2 phase cells. In SiHa cells, cell cycle experiments showed that SPOP knockdown could significantly promote the percentage of G1 phase cells and reduce the percentage of S phase cells. In ME-180 cells, cell cycle experiments showed that overexpression of SPOP could significantly reduce the proportion of G1 phase cells and increase the proportion of G2 phase cells. In MS751 cells, cell cycle experiments showed that overexpression of SPOP could significantly reduce the proportion of G1 phase cells and increase the proportion of S phase cells

To further evaluate whether SPOP affects cell proliferation, we performed a cell cycle assay. Knocking down SPOP significantly increased the percentage of HeLa cells in G1 phase and decreased the percentage of HeLa cells in S phase and G2 phase (Fig. 3A, B). In SiHa cells, the same effect was achieved for SPOP knockdown in G1 and S phase cells, except that there was no statistical significance in G2 phase cells (Fig. 3A, B). In the overexpressing cells, upregulated SPOP significantly decreased the percentage of ME-180 and MS751 cells in G1 phase, increased the percentage of ME180 cells in G2 phase and increased the percentage of MS751 cells in S phase (Fig. 3A, B). The above data suggest that SPOP can increase the proportion of S phase and G2 phase cells and reduce the proportion of G1 phase cells, thus promoting cell proliferation.

The above data demonstrated that SPOP can promote the proliferation of CC cells.

### SPOP promotes CC cell migration and invasion in vitro

**Fig. 4:**
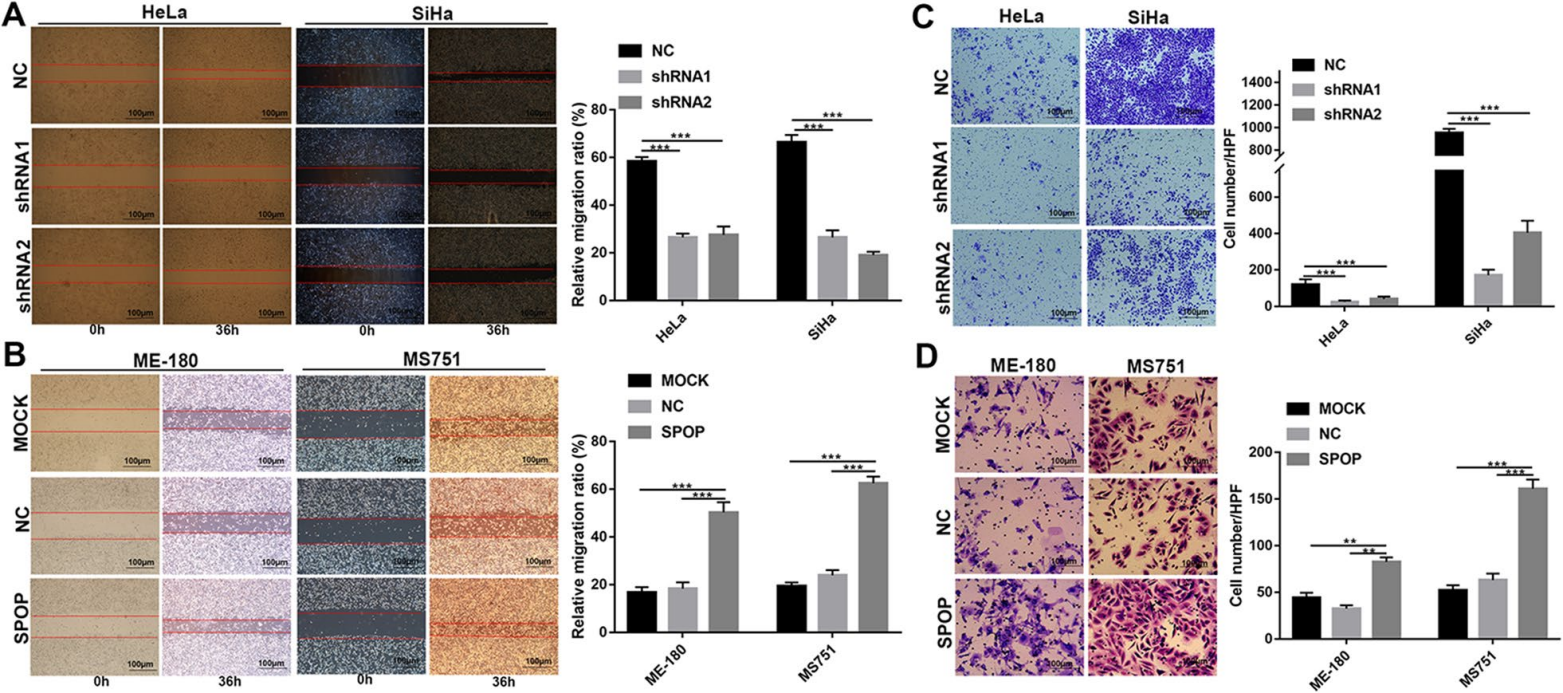
SPOP promotes CC cell migration and invasion in vitro. **A** Wound-healing assays showed that shRNA-mediated SPOP knockdown suppressed the migration of HeLa and SiHa cells. **B** SPOP overexpression significantly promoted ME-180 and MS751 cell migration. **C** The Transwell assay showed that invasion was suppressed when SPOP was silenced. **D** The promoting effect of SPOP on the invasion ability of ME-180 and MS751 cells was confirmed by SPOP overexpression

The relationship between SPOP expression and distant metastasis encouraged us to further study whether SPOP can affect cell migration. Our results of wound-healing assays showed that shRNA-mediated SPOP knockdown suppressed the migration of HeLa and SiHa cells (Fig. 4A) but that SPOP overexpression significantly promoted ME-180 and MS751 cell migration (Fig. 4B).

Moreover, the Transwell assay showed that invasion was suppressed when SPOP was silenced (Fig. 4C). Conversely, the promoting effect of SPOP on the invasion ability of ME-180 and MS751 cells was confirmed by SPOP overexpression (Fig. 4D). Overall, these data demonstrate that SPOP can promote CC cell migration and invasion.

### SPOP promotes CC cell proliferation and metastasis in vivo

**Fig. 5:**
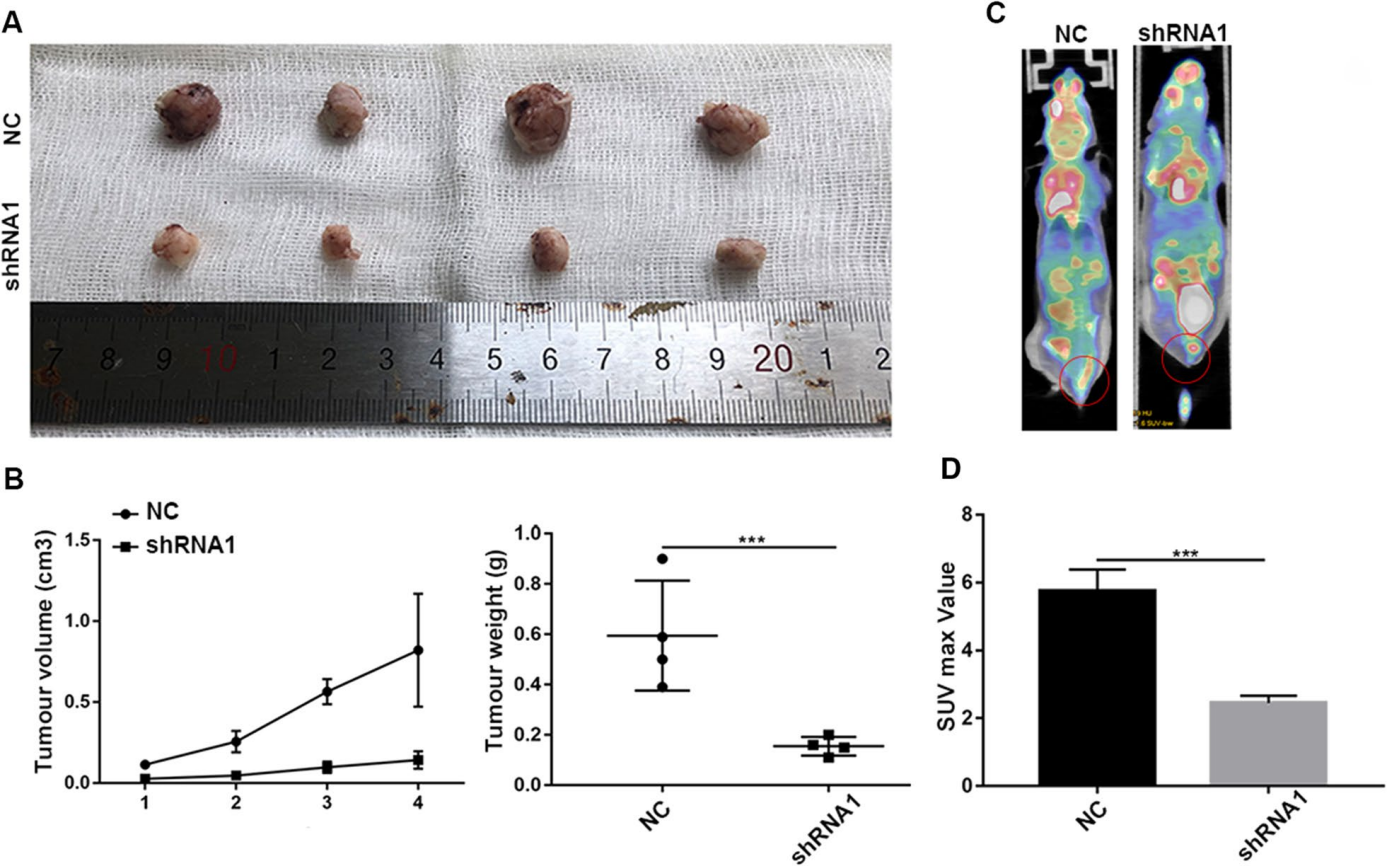
SPOP promotes CC proliferation and metastasis in vivo. **A** Xenografts were established in BALB/c nude mice by subcutaneously injecting SPOP-knockdown HeLa cells (shRNA1) or vector-expressing cells (NC). **B** Xenograft tumour growth curves of the shRNA1 and NC groups. Final tumour weights of xenograft tumours at sacrifice. **C** Micro-PET-CT imaging (the red circle indicates metastasis of CC). **D** SPOP knockdown markedly reduced F18-FDG uptake into the tumours in vivo

To further verify the effects of SPOP in vivo, HeLa cells with stable SPOP knockdown or empty vector transfection were injected subcutaneously into BALB/c nude mice or the vaginal wall. The volumes of the tumours that developed from SPOP knockdown HeLa cells were smaller than those of NC group (Fig. 5A, B). The average tumour weight was decreased in the knockdown group (Fig. 5B).

Additionally, F18-FDG PET-CT imaging measures the incorporation of the glucose analogue into tumours and is mainly used in the clinical diagnosis and staging of systemic metastasis of tumours. To determine whether SPOP modulates CC metastasis in vivo, we performed micro-PET-CT imaging of mice injected with SPOP-NC or SPOP-silenced CC cells. As a result, SPOP knockdown markedly reduced F18-FDG uptake into the tumours in vivo (Fig. 5C). The SUV max was 5.75 ± 0.45 in the NC group and 2.45 ± 0.15 in the knockdown group (NC group versus shRNA1 group, *P* = 0.02) (Fig. 5D). In summary, we conclude that SPOP can promote CC proliferation and metastasis in vivo.

### SPOP may promote migration by suppressing the spatial proximity between PD-1 and PD-L1

Using the HALO system, we analysed the number and spatial location of six immune targets on the TMA. We further divided the above data into two groups according to the expression of SPOP (IHC score 1: Low group; IHC score 2 and 3: High group), compared the differences in various immune infiltrations, and explored the potential mechanism of CC metastasis.

**Fig. 6:**
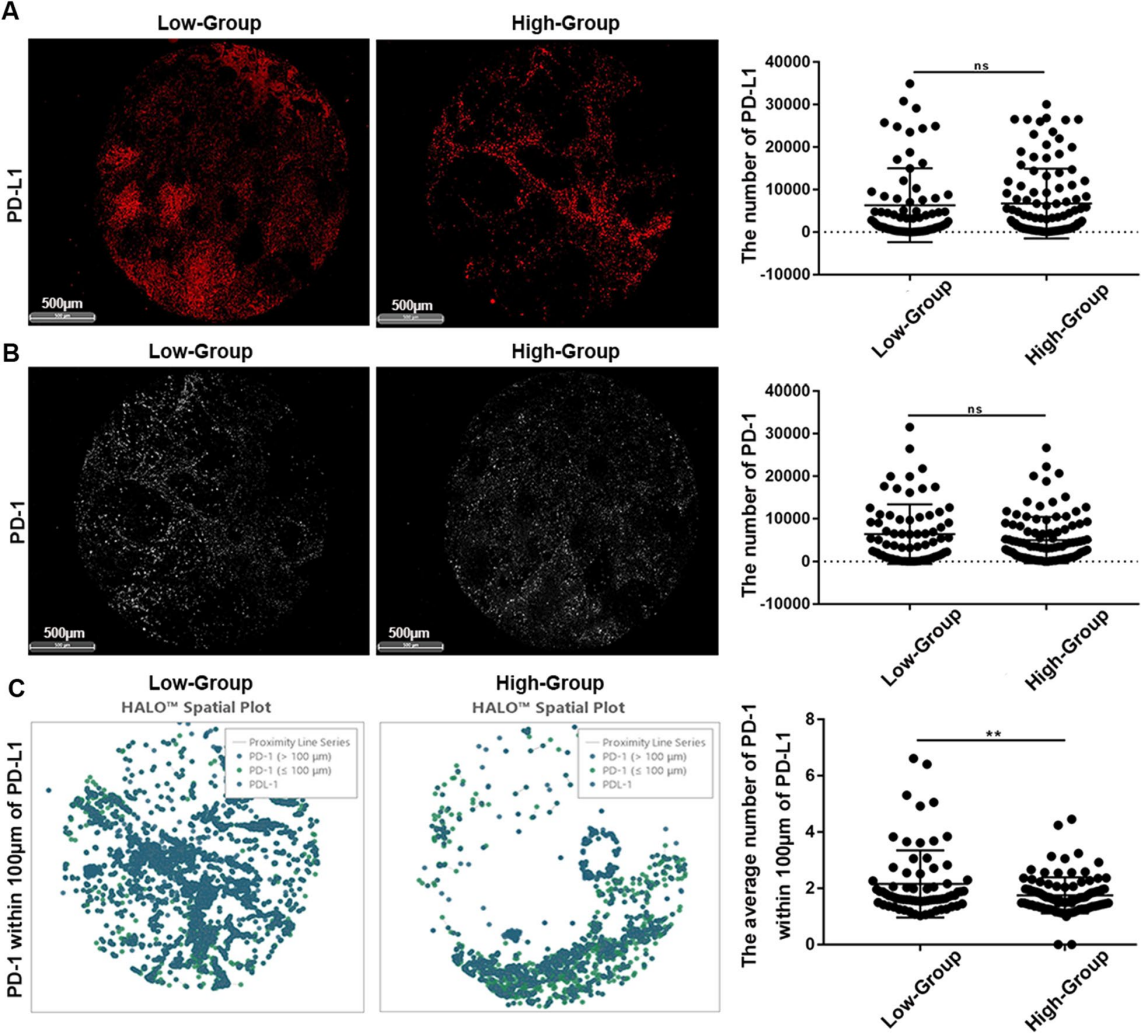
SPOP may promote migration by suppressing the spatial proximity between PD-1 and PD-L1. **A** SPOP did not affect PD-L1 expression or **B** PD-1 expression. **C** PD-1 was significantly farther from PD-L1 in spatial distance with increasing SPOP expression

PD-L1 expression was 6325 ± 1023 in the Low group and 6739 ± 808.3 in the High group (Low group versus High group, *P* = 0.75) (Fig. 6A). PD-1 expression was 6418 ± 822 in the Low group and 5009 ± 533.8 in the High group (Low group versus High group, *P* = 0.13) (Fig. 6B). The difference between the two groups was not statistically significant, suggesting that SPOP did not affect PD-L1 or PD-1 expression. However, the average number of PD-1 within 100 μm of PDL-1 was 2.56 ± 0.14 in the Low group and 1.75 ± 0.063 in the High group (Low group versus High group, *P* < 0.01) (Fig. 6C).

In summary, PD-1 was significantly farther from PD-L1 in terms of spatial distance with increasing SPOP expression. Previously, we verified that SPOP can promote the migration and invasion of CC cells in vitro. Therefore, we speculated that SPOP could promote CC metastasis by keeping PD-1 away from PD-L1 in terms of spatial distance. This may be the potential mechanism by which SPOP promotes CC metastasis.

### SPOP can promote the immune tolerance of PD-1 in vitro

**Fig. 7:**
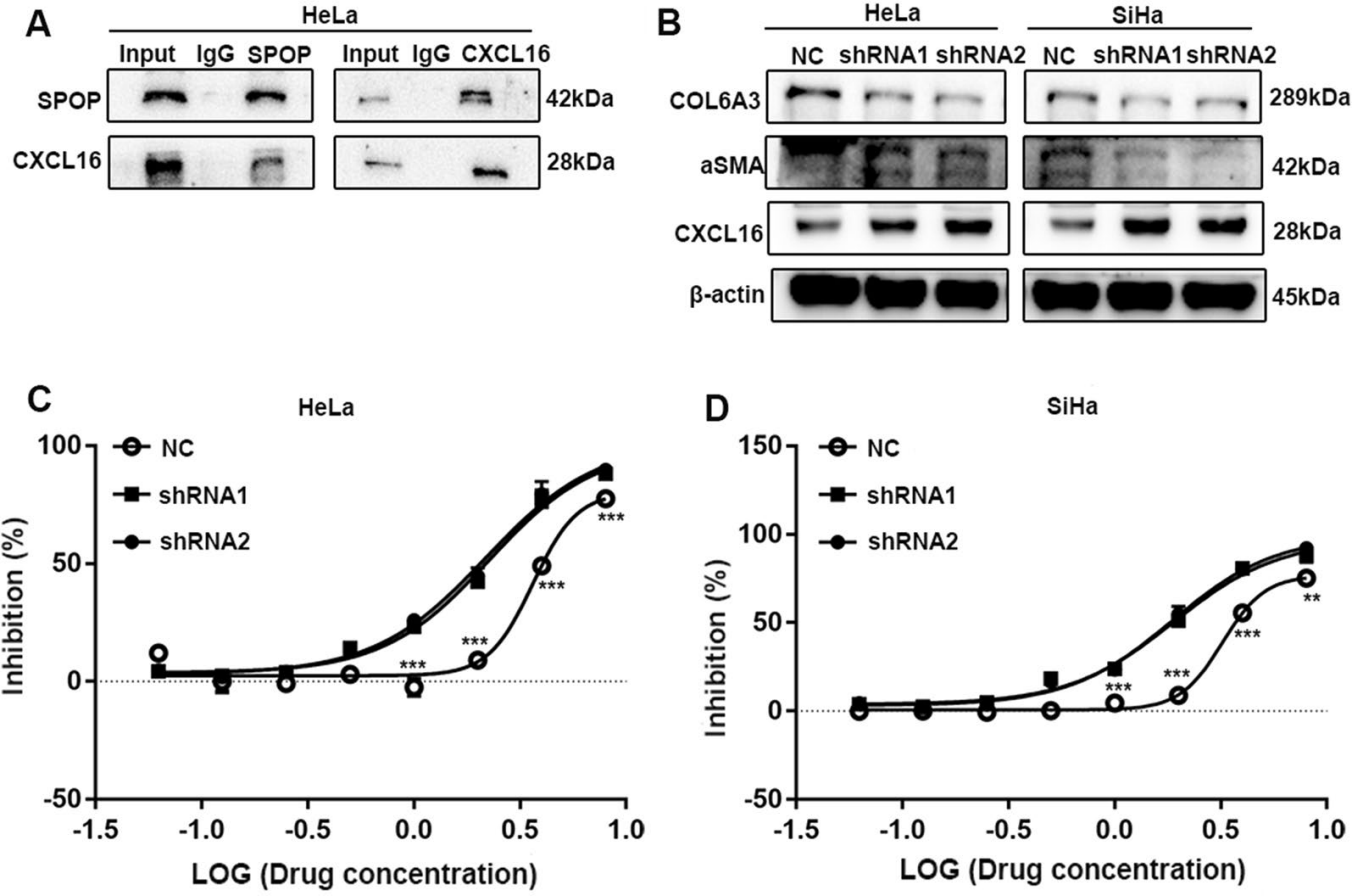
SPOP can promote the immune tolerance of PD-1 in vitro. **A** The results of Co-IP.** B** Knocking down SPOP reduces the expression of the CAF markers ⍺SMA and COL6A3, and increases CXCL16 expression. **C** The figure suggests that SPOP can promote PD-1 resistance in HeLa or **D** SiHa cells

Wang et al. revealed chemokine receptor-ligand interactions within and across compartments mediating immune infiltration [57]. CXCL16 is a chemokine that is produced on tumour cells, particularly infiltrating tumour cells, and is known to signal through the CXCR6 receptor [58]. CXCL16 can also recruit CXCR6 + CD8 + T cells in a less immunogenic tumour model, according to Matsumura et al. [59]. The chemotaxis effect of CXCL16 varies between excluded and invasive cancers. Additionally, the CXCL16-CXCR6 axis represent a crucial factor contributing to the tumour continuum in ovarian cancer (OC) [57]. Here, we demonstrated that SPOP can bind to CXCL16 (Fig. 7A) and that knocking down SPOP can boost CXCL16 expression (Fig. 7B).

The development of unique cancer-associated fibroblasts (CAFs) in the stroma in excluded tumours creates a physical barrier that prevents T lymphocytes from communicating with tumour cells [60]. We also found that knocking down SPOP reduces the expression of the CAF markers αSMA and COL6A3 (Fig. 7B) [61]. This shows that SPOP can increase the spatial distance between T cells and tumour cells by promoting the development of CAFs. As a result, we show that SPOP can enhance its own degradation by binding CXCL16 and encouraging the creation of CAFs, increasing the geographical distance between T cells and tumour cells.

In this section, we discuss how SPOP can support the function of PD-1 in immune tolerance. The cells in the control group and SPOP knockdown group were treated with different concentrations of PD-1, and the change in IC50 indicated that SPOP can promote the spatial distance of PD-1 and PD-L1 to achieve immune tolerance. In HeLa cells, the IC50 reached 3.63 ± 0.22 in the NC group, 2.26 ± 0.08 in the shRNA1 group, and 2.18 ± 0.07 in the shRNA2 group (NC versus shRNA1, *P* < 0.05; NC versus shRNA2, *P* < 0.05) (Fig. 7C). The above data suggest that SPOP can promote PD-1 resistance in HeLa cells. In SiHa cells, the IC50 reached 3.217 ± 0.17 in the NC group, 1.901 ± 0.07 in the shRNA1 group, and 1.869 ± 0.06 in the shRNA2 group (NC versus shRNA1, *P* < 0.05; NC versus shRNA2, *P* < 0.05) (Fig. 7D). The above data suggest that SPOP can promote PD-1 resistance in SiHa cells.

In conclusion, in vitro experiments have demonstrated that SPOP can promote the separation of PD-L1 from PD-1 in a spatial position by binding PD-L1, thus achieving immune tolerance and promoting the progression of CC.

### SPOP can promote PD-1-mediated immune tolerance via CXCL16

**Fig. 8:**
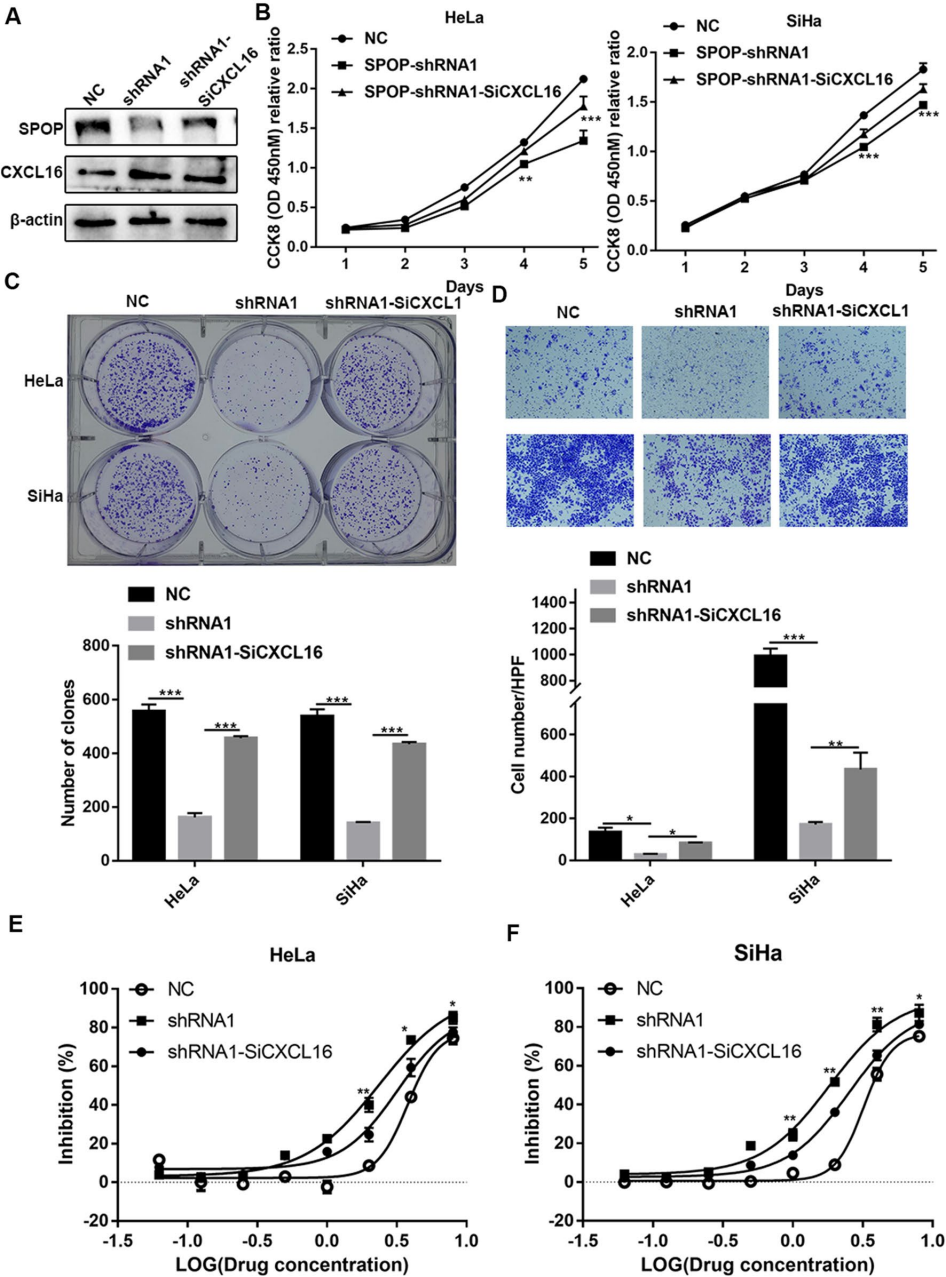
SPOP can promote the immune tolerance of PD-1 via CXCL16. **A **CXCL16 transient siRNA-knockdown in HeLa cells with SPOP knockdown did not increase SPOP expression, but only reduced the increased-CXCL16 expression. **B**–**D** The results of CCK-8, colony formation and invasion assays showed that CXCL16 knockdown could reverse these phenotypes. **E**, **F** The IC50 results showed that CXCL16 knockdown could functionally reverse immune tolerance

To further explore whether SPOP mediates PD-1 immune tolerance through CXCL16, CXCL16-related rescue experiments were conducted. This will further strengthen the logic of SPOP-mediated immune tolerance of PD-1. This part of the rescue experiments was still based on the proliferation, metastasis phenotype and functional PD-1 inhibition rate of tumour cells as described above. Firstly, we found that transient siRNA-mediated knockdown of CXCL16 in HeLa cells with SPOP knockdown did not increase SPOP expression but only attenuated the increase in CXCL16 expression (Fig. 8A). This cell line was then used for CCK-8, colony formation and invasion assays, and the results showed that CXCL16 knockdown could reverse the related phenotypes (Fig. 8B–D). The IC50 results further showed that CXCL16 knockdown could functionally reverse immune tolerance (Fig. 8E, F). In summary, SPOP can mediate PD-1 immune tolerance through CXCL16.

## Discussion

### Main findings and interpretation

Previous studies have demonstrated that SPOP can suppress or promote tumorigenesis in a variety of malignancies, including lung, colon, gastric, prostate, and liver cancers [10]. However, few studies have focused on SPOP in the development of CC, and only two reports have also shown dual effects [17, 18]. Traditional procedures have been used to elucidate the mechanism by regularly exploring such molecular pathways that would destroy the spatial structure [62, 63]. By the m-IF and HALO systems, the spatial orientation interrelation of immune cells and immune markers would be preserved [22, 64]. Through a series of experiments, we demonstrated for the first time that SPOP promotes CC pLN metastasis by promoting PD-1 movement away from PD-L1.

Beginning with this study, we found that the expression of SPOP was increased in CC in the pLN-positive group compared with the pLN-negative group. This suggests that SPOP may be associated with pLN metastasis in CC. Second, further analysis found that the High-SPOP group had poorer OS and RFS. In conclusion, these results demonstrated that SPOP is upregulated in CC with pLN metastasis and negatively associated with patient outcomes.

Next and most importantly, we needed to conduct in vitro or in vivo experiments to prove a causal relationship between SPOP and CC cell migration and invasion. We showed in vitro that knockdown or overexpression of SPOP can significantly inhibit or promote, respectively, CC cell proliferation, cloning, cell cycle, wound healing, and Transwell assays. These data suggest that SPOP can promote the migration and invasion of CC cells. Moreover, our in vivo experiments demonstrated that SPOP knockdown can significantly inhibit the metastasis of CC cells.

From bench to bedside, we analysed the immune network environment of the CC TMA again to determine the potential mechanism of pLN metastasis. PD-L1 expression has been detected in the majority of CC using IHC analysis of tumour cells [65], suggesting that anti-PD-1 therapies may be effective in CC. As a result, a series of clinical trials for the anti-PD-1-based treatment of advanced or recurrent CC have emerged, such as Keynote 028, Keynote 158, and Checkmate 358 [66–68]. We can conclude that pembrolizumab (an anti-PD-1 drug) provides durable antitumor activity in partial patients, regardless of PD-L1 expression, and has manageable toxicity. Afterwards, pembrolizumab received approval from the Food and Drug Administration (FDA) for the treatment of patients with recurrent or metastatic CC with disease progression during or after chemotherapy (whose tumours express PD-L1) based on the objective response rate and durability of responses [67]. However, the response rate of PD-1 in tumors is still low in general, which requires further study. Here, we have discovered a new mechanism of immune checkpoint inhibitors (ICIs) in the treatment of CC. Through quantitative pathology and tissue space analysis of the HALO system, the tumour area, PD-1 numbers, and PD-L1 numbers of each point on TMA representing tissue samples from different patients were presented. According to statistical principles, if continuous variables with more than 30 cases conform to the normal distribution, *t* test can be used to analyse the differences between the two groups; our analysis showed that the differences were not statistically significant [69]. However, the number of PD-1 molecules in the 100 µm PD-L1 range decreased significantly with increasing SPOP expression. Therefore, the core idea of the whole paper also emerged. With the increase in SPOP expression, PD-1 was significantly farther away from PD-L1 in terms of spatial distance.

Lastly, to further verify the causal relationship between the PD-1/PD-L1 axis and SPOP, a Co-IP-MS experiment was conducted. We dissected how the cellular components of the tumour, immune compartments interact through chemical-receptor signaling and spatial barrier levels. It has been previously reported that tumour cells can potentially mediate T cell recruitment via the CXCL16-CXCR6 axis [57, 58, 70]. In addition, CAFs can create a physical barrier that blocks the access of T cells to the tumour epithelium [60, 61, 71]. Our study revealed that SPOP can bind CXCL16 and promote its degradation, resulting in CAFs increasing the spatial distance between immune cells and tumour cells. Moreover, we compared the control group and SPOP knockdown group with different concentrations of PD-1 in HeLa and SiHa cell lines in vitro and found that knocking down SPOP could significantly reduce the IC50 value of PD-1. Further knockdown of CXCL16 in SPOP-shRNA1 cell lines could reverse these effects.

These results suggest that SPOP can achieve immune tolerance by promoting PD-1 sequestration from PD-L1.

### Limitations

Further applications such as mRNA delivery by nanoliposomes should be used to validate these results in clinical trials to translate these basic science findings to the clinical setting.

## Conclusion

**Fig. 9:**
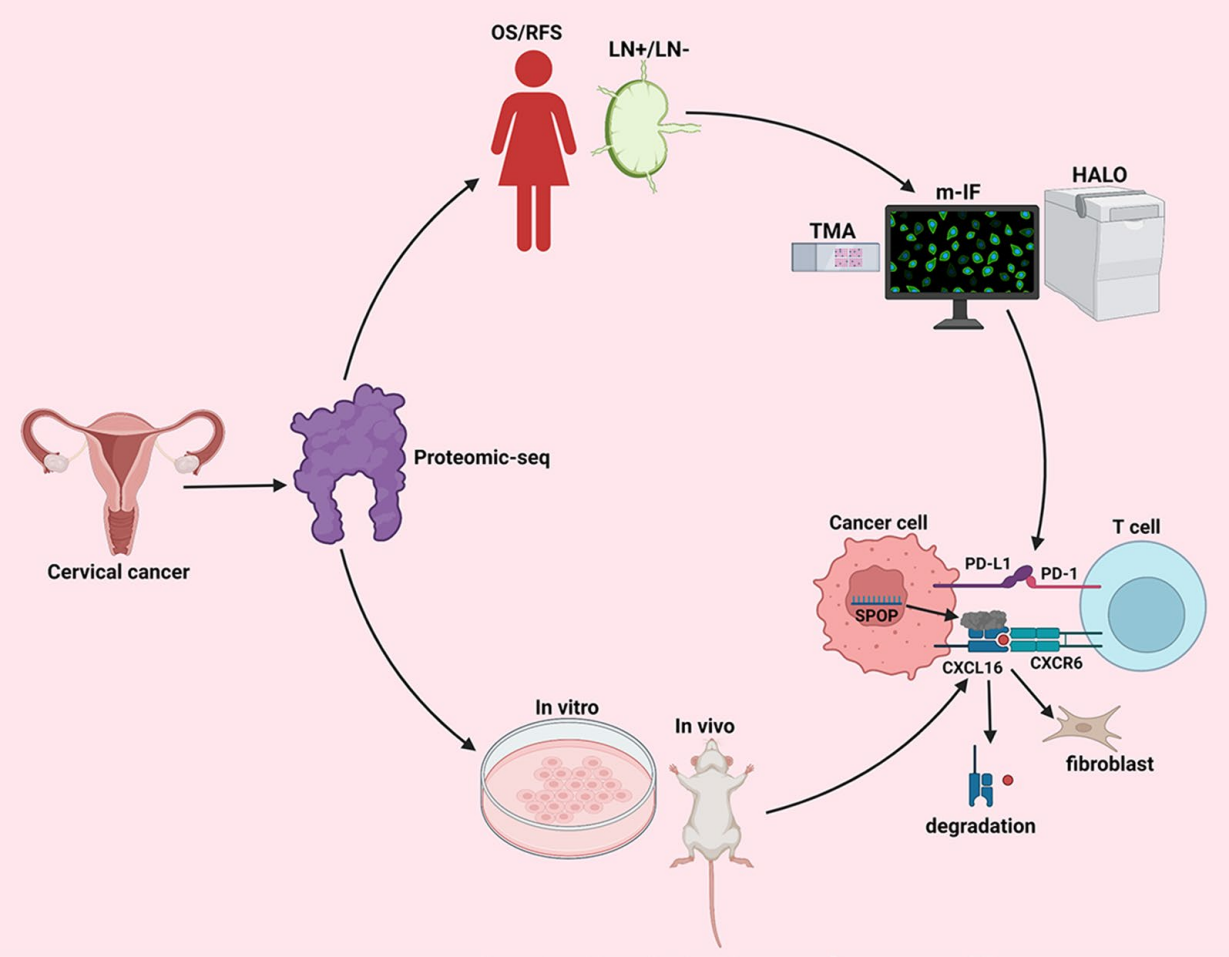
Flow diagram

In conclusion, SPOP can inhibit the immune microenvironment by promoting the movement of PD-1 away from PD-L1, thereby promoting pLN metastasis of CC, resulting in worse OS and RFS in patients. This is a typical process of deep clinical and basic cross-fusion validation exploration. Our study began with clinical tissue data, proved causality in vitro and in vivo, and then returned to clinical samples to explore the potential causes of pLN metastasis through the spatial location relationship from m-IF, finally performing validation in vitro (Fig. 9). This will expand our understanding of CC progression and shed light on therapeutic targets for CC.

## Data Availability

The data that support the results of this study are available from the corresponding author upon reasonable request.
